# Incidence of peripheral intravenous catheter phlebitis and its associated factors among patients admitted to University of Gondar hospital, Northwest Ethiopia: a prospective, observational study

**DOI:** 10.1186/s12959-021-00301-x

**Published:** 2021-07-13

**Authors:** Mulugeta Lulie, Abilo Tadesse, Tewodros Tsegaye, Tesfaye Yesuf, Mezgebu Silamsaw

**Affiliations:** Department of Internal Medicine, School of Medicine, College of Medicine and Health Sciences, University of Gondar, Gondar, Ethiopia

**Keywords:** Peripheral intravenous catheters, Phlebitis, Northwest Ethiopia

## Abstract

**Background:**

Phlebitis, inflammation of tunica intima of venous wall, occurred in 13–56% of hospitalized patients. It is characterized by pain, erythema, swelling, palpable venous cord, and pussy discharge at catheter site. Cannula-related blood stream infection (CRBSI) is recognized complication of phlebitis. Adverse outcomes of phlebitis embrace patient discomfort, longer hospital stay and higher health care cost. This study aimed to determine the incidence and associated factors of peripheral vein phlebitis among hospitalized patients.

**Methods:**

A hospital-based prospective, observational study was conducted between April 1 and August 31, 2020 at University of Gondar hospital, Northwest Ethiopia. A consecutive sampling method was used to recruit 384 patients. Patients were interviewed to obtain socio-demographic data. Relevant medical history and laboratory parameters were obtained from patients’ records. Presence and severity of phlebitis was identified by Jackson’s Visual Infusion Phlebitis (VIP) Scoring System. The Data were entered into EPI Info version 4.4.1 and transported to SPSS version 20 for analysis. Logistic regression analysis was used to identify associated factors with occurrence of phlebitis. P-value < 0.05 was used to declare significant association.

**Result:**

A total of 384 study subjects were included in the study. The mean age of study subjects was 46 years, with a range of 19 to 96 years. The incidence of phlebitis was 70% among study subjects. Mid-stage (grade 3) and advanced-stage (grade 4) phlebitis were noticed in 136/268 (51%) and 89/268 (33%) respectively. Odds of developing phlebitis were twofold higher in patients with catheter-in situ > 96 h (AOR = 2.261, 95% CI 1.087–4.702, P-value = 0.029) as compared to those with catheter dwell time < 72 h. Female patients were 70% (AOR = 0.293, 95% CI 0.031–0.626, P-value = 0.002) lower than male patients with risk of developing phlebitis. Patients who use infusates were 53% (AOR = 0.472, 95% CI 0.280–0.796, P-value = 0.005) less likely to develop phlebitis as compared to those who didn’t use infusates.

**Conclusion:**

The cannula must be reviewed on daily basis, and it should be removed if it stayed later than 96 h.

## Background

Peripheral intra venous catheter (PIVC) insertion is the most frequently performed procedure in hospital settings. Approximately 33–67% of hospitalized patients require at least one peripheral vein insertion. Peripheral vein catheters are required for administration of intravenous drugs, infusate solutions, blood products and parenteral feeding. It is as well necessary for access to vascular procedures [1–13]. Despite PIVC benefits, its use is not without potential complications such as phlebitis, infiltration, extravasation, occlusion and dislodgment [3, 5–10, 14–16]. Peripheral vein phlebitis occurred in 13–56% of hospitalized patients [7–10, 15, 16]. Phlebitis is clinically manifested by pain, erythema, swelling, palpable venous cord, and pussy discharge at catheter site [4–8, 10, 11, 15–17]. Cannula-related blood stream infection (CRBSI) is recognized complication of phlebitis. Presence and severity of phlebitis is evaluated by Jackson’s Visual Infusion Phlebitis Scoring System [17]. Adverse outcomes of Phlebitis embrace patient discomfort, longer hospital stay and higher health care cost [6, 9–13]. Factors contributing to occurrence of phlebitis include mechanical, chemical, biological, patient, and health practice-related factors [7–13, 15, 16]. Mechanical factors consist of cannula size, site of catheter placement, catheter dwell time and type of catheter (Teflon Vs Vialon). Teflon catheter type, large cannula size, near joint-catheter placement, and catheter dwell time > 96 h predispose to phlebitis. Type of intravenous drugs (irritant, vesicant) and solution characteristics (PH^+^, osmolality) are components of chemical factors. Irritant intravenous drugs and hyperosmolar infusate solutions cause vascular endothelial injury, and results in phlebitis. Biological factors embrace bacterial colonization, biofilm formation and infection. Patient-related factors take account of age, gender, nutritional status, immunosuppression and co-existing comorbidities. Those with malnutrition, immunosuppression, co-morbidities, and elderly (age > 65 years) are vulnerable to phlebitis. Implementing aseptic precautions and health professional skill on catheter securement are the frequently implicated health practice-related factors. Poor aseptic technique and improperly securing of cannula are among listed causes of phlebitis.

This study was the first of its kind in Ethiopia to determine the incidence and associated factors of peripheral vein phlebitis among adult hospitalized patients.

## Methods

### Study design and setting

A hospital-based prospective, observational study was conducted between April 1 and August 31, 2020 at emergency unit, medical and surgical wards of University of Gondar hospital. Both wards had a capacity of 92 beds. The hospital is located in Northwest Ethiopia, which is 750 km away from the capital, Addis Ababa. The hospital had a catchment population of 5 million people.

### Study subjects and variables

#### Study subjects

All patients admitted to emergency unit, medical and surgical wards during the study period were considered as study population. Patients older than 18 years old, who were admitted to emergency unit, medical and surgical wards and who were on peripheral intravenous catheter, were included in the study. Patients with history of allergy to intravenous cannula, or those unable to give consent were excluded from the study.

#### Study variables

Dependent variables: Peripheral intravenous catheter (PIVC) phlebitis.

Independent variables: (1) Socio-demographic characteristics include age, gender, occupation, marital status, educational level, income level, residence and religion (2) Clinical characteristics include admission diagnosis, duration of hospital stay, cannula insertion site, catheter size (gauge), catheter dwell time, intravenous drugs use, infusates use, blood products use, performance level, and co-existing comorbidities.

### Sample size and sampling procedure

The sample size was calculated using single population proportion formula with the assumption of 95% confidence level, 5% margin of error, and taking 50% estimated proportion of phlebitis. Consecutive sampling method was used to recruit 384 study subjects.

### Data collection instrument and procedures

Data were collected through an investigator administered pre-designed questionnaire. Patients were interviewed to obtain socio-demographic data. Relevant medical history and laboratory parameters were obtained from patients’ records. Three investigators (one medical doctor, two clinical nurses) participated in the data collection process and follow-up of patients on indwelling catheter. Insertion of a catheter was performed by clinical nurses. Hygienic hand-rub with 70% alcohol was done after hand washing. Patient’s skin was disinfected using 1% iodine solution and 70% alcohol. Using clean gloves, a catheter was inserted into a peripheral vein of a patient. The catheter was fixed to the patient’s skin with protective dressing and plaster. Patients on indwelling catheter were followed daily by investigators from insertion to developing phlebitis or catheter removal. Those who developed phlebitis were graded based on Jackson’s Visual Infusion Phlebitis (VIP) Scoring System [17] and were taken care of as per the recommendations of Infusion Therapy Standard of Practice [2].

### Data analysis

Data were entered into EPI Info version 4.4.1 and transported to SPSS version 20 for analysis.

Patient characteristics were reported as counts (percentages) for categorical variables, and mean with standard deviation for continuous variables. Bi-variable and multi-variable logistic regression models were constructed to identify independently associated factors with occurrence of phlebitis. Those variables with a P-value < 0.25 in the bi-variable analysis were exported to multi-variable analysis to control the possible effect of confounders. Crude odds ratio (COR) and adjusted odds ratio (AOR) were reported. P-value < 0.05 was used to declare significant association.

### Ethical considerations

The research protocol complied with Declaration of Helsinki, and was approved by local ethics committee (06/09/2012; IRB No. 09/26/629/12). Study subjects were recruited only after informed written consent was obtained. All data obtained were treated confidentially.

### Definition of terms

#### Phlebitis

Occurrence of at least one inflammatory sign or symptom related to the catheter insertion, comprising persistent pain (duration longer than 2 h after end of infusion) and/or erythema and/or swelling (induration) and/or palpable cord and/or pussy discharge at intravenous site and/or pyrexia [17].

#### Grading of phlebitis

Grade 1 phlebitis: one of the following is evident: Pain near IV site or erythema near IV site; Grade 2 phlebitis (early stage of phlebitis): Two of the following are evident: Pain at IV site, erythema or swelling; Grade 3 phlebitis (mid stage of phlebitis): All of the following signs are evident: Pain along path of cannula, erythema and induration; Grade 4 phlebitis (advanced stage of phlebitis or early stage of thrombophlebitis): All of the following signs are evident and extensive: Pain along path of cannula, erythema, induration and palpable venous cord; Grade 5 phlebitis (Advanced stage of thrombophlebitis): All of the following signs are evident and extensive: Pain along path of cannula, erythema, induration, palpable venous cord, and visible pussy discharge at IV site or pyrexia [17].

## Result

### Socio-demographic characteristics of study participants

A total of 384 participants were included in the study. The mean age of study subjects was 46 years, with a range of 19 to 96 years. More than half (52%) of them were males and were rural dwellers (53%). Majority (89%) of respondents were Christian by religion. Fewer than half (43%) of study subjects attended formal education (Table 1).

**Table 1:** Socio-demographic characteristics of admitted patients with PIVC phlebitis at University of Gondar hospital, Northwest Ethiopia, April 1, 2020 to August 31, 2020

Variables	Frequency (No.)	Percentage (%)
Age (years)		
15–40	169	44.0
40–60	125	32.6
> 60	90	23.4
Gender		
Male	199	51.8
Female	185	48.2
Religion		
Christian	343	89.3
Muslim	41	10.7
Residence		
Urban	179	46.6
Rural	205	53.4
Marital status		
Single	69	18.0
Married	247	64.3
Divorced	32	8.3
Widowed	36	9.4
Educational level		
Unable to read and write	167	43.5
Able to read and write	51	13.3
Alimentary school	67	17.4
Secondary school	49	12.8
College and above	50	13.0
Occupation		
Farmer	136	35.4
Government employee	52	13.5
Merchant	33	8.6
Housewife	104	27.1
Daily laborer	21	5.5
Student	25	6.5
Others	13	3.4
Income level (birr)		
< 1500	166	43.2
1500–2500	119	31.0
2500–3500	35	9.1
> 3500	64	16.7

*NB* Others * jobless, *PIVC* peripheral intravenous catheter

### Clinical characteristics of study participants

Two-thirds (63%) of the patients were semi-ambulatory. Majority of patients were hospitalized with lung (25%), heart (18%) and CNS (17%) diseases. Two-third (63%) of patients presumed to have bacterial infections. Fewer than 10% of patients each were diagnosed to have diabetes, CKD, HIV/AIDS, and malignancy as co-morbidities. Indications for PIVC insertion were administration of intravenous drugs (73%), infusates (65%), and blood products (14%). Half (53%) of the catheters were inserted in emergency situations. Forearm was used as catheter placement in half (52%) of patients. 20 G sized cannula was used in most (81%) patients. Two-third (66%) of patients had PIVC in-situ for 96 h or less (Tables 2 and 3).

**Table 2:** Clinical characteristics of admitted patients with PIVC phlebitis at University of Gondar hospital, Northwest Ethiopia, April 1, 2020 to August 31, 2020

Variables	Frequency (No.)	Percentage (%)
Admission diagnosis		
Lung disease	97	25.3
Heart disease	68	17.7
CNS disease	65	16.9
Chronic liver disease	21	5.5
Chronic kidney disease	16	4.1
Hematological disorders	18	4.7
Fracture	13	3.4
Others	86	22.4
Performance level on admission		
Ambulatory	68	17.7
Semi-ambulatory	241	62.8
Bedridden	75	19.5
Duration of hospital stay (days)		
< 3	26	6.8
3–7	120	31.2
7–14	161	41.9
> 14	77	20.1
Procedure place		
Emergency unit	203	52.9
Wards	181	47.1
Catheter dwell time (days)		
< 3	171	31.3
3–4	83	21.7
> 4	130	33.9
Cannula insertion site		
Dorsum of hand	148	38.5
Forearm	201	52.3
Antecubital fossa	35	9.2
Catheter size (gauge)		
18	61	15.9
20	311	81.0
22	8	2.1
24	4	1.0
IV drugs use		
Yes	282	73.4
No	102	26.6
Infusates use		
Yes	251	65.4
No	133	34.6
Blood products use		
Yes	52	13.5
No	332	86.2

*NB* Others * Osteomyelitis, Bowel obstruction, *PIVC* peripheral intravenous catheter, *IV* intravenous

**Table 3:** Co-morbidities among admitted patients with PIVC phlebitis at University of Gondar hospital, Northwest Ethiopia, April 1, 2020 to August 31, 2020

Variables	Frequency (No.)	Percentage (%)
Diabetes		
Yes	38	9.9
No	346	90.1
Hypertension		
Yes	89	23.2
No	295	76.8
HIV/AIDS		
Yes	24	6.3
No	159	41.4
Unknown	201	52.3
Chronic kidney disease		
Yes	30	7.8
No	354	92.2
Malignancy		
Yes	30	7.8
No	354	92.2
Concomitant bacterial infections		
Yes	243	63.3
No	141	36.7

*NB* AIDS, acquired immune deficiency syndrome, *HIV* human immuno deficiency virus, *PIVC* peripheral intravenous catheter

### Incidence and grades of phlebitis

**Fig. 1:**
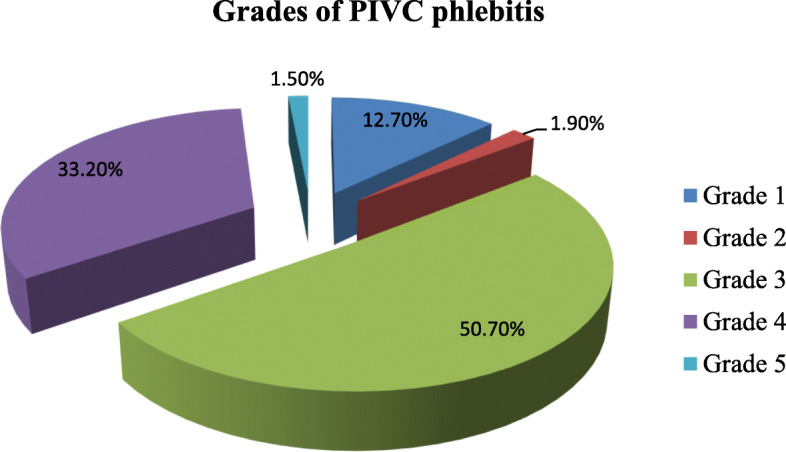
Grades of peripheral intravenous catheter (PIVC) phlebitis

The incidence of phlebitis was 70% (268/384) among study subjects. Among those who developed phlebitis, mid-stage (grade 3) and advanced-stage (grade 4) phlebitis were noticed in 136/268 (51%) and 89/268 (33%) respectively. Advanced stage thrombophlebitis (grade 5) occurred in 4/268 (1.5%) of phlebitis cases (Fig. 1).

### Factors associated with occurrence of phlebitis

Age, gender, residence, religion, educational level, admission diagnosis, duration of hospital stay, catheter dwell time, intravenous drugs use, infusates use, and co-existing comorbidities such as HIV/AIDS and hypertension were identified as predictors of phlebitis on bi-variable analysis. When variables in bi-variable analysis with P-value < 0.25 were regressed in multi-variable analysis; only gender, catheter dwell time and infusates use were independently associated with occurrence of phlebitis. Odds of developing phlebitis were twofold higher in patients with catheter-in situ > 96 h (AOR = 2.261, 95% CI 1.087–4.702, P-value = 0.029) as compared to those with catheter dwell time < 72 h. Female patients were 70% (AOR = 0.293, 95% CI 0.137–0.626, P-value = 0.002) lower than male patients with risk of developing phlebitis. Patients who used infusates were 53% (AOR = 0.472, 95% CI 0.280–0.796, P-value = 0.005) less likely to develop phlebitis as compared to those who didn’t use infusates (Table 4).

**Table 4:** Bi-variable and multi-variable logistic regression analysis of factors associated with PIVC phlebitis among admitted patients at University of Gondar hospital, Northwest Ethiopia, April 1, 2020 to August 31, 2020

Variables	Phlebitis		COR (CI)	P-value	AOR (CI)	P-value
	Yes	No				
Age (years)						
18–40	42	127	1		1	
40–60	43	82	1.59 (0.91–2.77)	0.104	1.19 (0.55–2.58)	0.650
> 60	31	59	1.02 (0.57–1.77)	0.995	0.89 (0.45–1.75)	0.730
Gender						
Male	128	71	1		1	
Female	140	454	0.58 (0.37–0.90)	0.016	0.29 (0.13–0.63)	0.002
Residence						
Urban	119	60	1		1	
Rural	149	56	0.75 (0.48–1.15)	0.187	0.65 (0.33–1.28)	0.209
Religion						
Christian	246	97	1		1	
Muslim	23	18	2.07 (1.06–4.02)	0.033	1.86 (0.71–4.91)	0.210
Educational level						
Unable to read and write	126	41	1		1	
Able to read and write	28	23	1.09 (0.54–2.20)	0.817	1.53 (0.47–5.02)	0.483
Elementary school	44	23	0.47 (0.21–1.08)	0.077	0.77 (0.23–2.62)	0.680
Secondary school	37	12	0.74 (0.34–1.65)	0.467	1.04 (0.33–3.31)	0.942
College and above	36	14	1.19 (0.49–2.94)	0.692	1.86 (0.59–5.75)	0.284
Admission diagnosis						
Lung disease	69	28	1			
Heart disease	42	26	0.90 (0.47–1.72)	0.749	1.3 (0.6–2.81)	0.511
CNS disease	47	18	0.59 (0.3–1.17)	0.130	0.75 (0.33–1.70)	0.488
Liver disease	17	4	0.95 (0.46–1.97)	0.897	1.32 (0.56–3.09)	0.522
Renal disease	8	8	1.55 (0.47–5.10)	0.469	1.74 (0.48–6.34)	0.404
Hematological diseases	12	6	1.22 (0.31–4.82)	0.78	1.19 (0.26–5.46)	0.826
Fracture	10	3	0.37 (0.12–1.09)	0.07	0.45 (0.12–1.64)	0.225
Others	63	23	0.57 (0.25–2.17)	0.572	1.3 (0.37–4.59)	0.688
Co-morbidities						
Diabetes						
Yes	26	12	1.07 (0.52–2.21)	0.85		
No	242	104	1			
Hypertension						
Yes	57	32	0.71 (0.43–1.17)	0.179	1.08 (0.57–2.05)	0.804
No	211	84	1		1	
HIV/AIDS						
Yes	12	12	1		1	
No	115	44	0.43 (0.18–1.00)	0.050	0.56 (0.20–1.58)	0.275
Unknown	141	60	1.11 (0.70–1.76)	0.651	1.22 (0.71–2.11)	0.488
CKD						
Yes	20	10	1.17 (0.53–2.58)	0.69		
No	250	104	1			
Malignancy						
Yes	22	8	1.07 (0.50–2.54)	0.64		
No	248	106	1			
Concomitant bacterial infections						
Yes	168	75	1.09 (0.69–1.72)	0.71		
No	100	41	1			
Duration of hospital stay						
< 3 days	19	7	1		1	
3–7 days	89	31	1.47 (0.55–3.93)	0.447	1.75 (0.56–5.48)	0.338
7–14 days	110	51	1.55 (0.83–2.89)	0.167	1.73 (0.85–3.53)	0.131
> 14 days	50	27	1.17 (0.66–2.07)	0.603	1.21 (0.63–2.32)	0.563
Place of procedure						
Emergency unit	146	57	1			
Wards	122	59	0.60 (0.07–5.70)	0.65		
Catheter dwell time						
< 3 days	119	52	1		1	
3–4 days	66	17	1.3 (0.8–2.10)	0.294	1.21 (0.68–2.15)	0.511
> 4 days	83	47	2.2 (1.16–4.18)	0.016	2.26 (1.09–4.70)	0.029
Cannula insertion site						
Dorsum of hand	95	53	0.85 (0.61–1.24)	0.422		
Forearm	144	57	0.91 (0.55–2.20)	0.652		
Antecubital fossa	29	6	1			
Cannula size (gauge)						
18	4120	1				
20	218	93	1.00 (0.63–1.98)	1.000		
22	6	2	0.78 (0.16–3.94)	0.761		
24	3	1	0.68 (0.13–3.69)	0.660		
Intravenous drugs use						
Yes	202	80	0.73 (0.45–1.18)	0.193	0.88 (0.49–1.59)	0.682
No	66	36	1		1	
Intravenous infusates use						
Yes	189	62	0.48 (0.31–0.75)	0.001	0.47 (0.28–0.79)	0.005
No	79	54	1		1	
Blood products use						
Yes	37	15	0.93 (0.49–1.77)	0.82		
No	231	101	1			

*NB* AIDS, acquired immune deficiency syndrome, *AOR* adjusted odds ratio, *COR* crude odds ratio, *CI* confidence interval, *CKD* chronic kidney disease, *HIV* human immune deficiency virus, *PIVC* peripheral intravenous catheter

Place of procedure done, catheter site placement, catheter size (gauge), use of blood products, concomitant bacterial infections, and co-existing comorbidities such as diabetes, CKD and malignancy were not found to be significantly associated with occurrence of phlebitis in bi-variable analysis (P-value > 0.25).

## Discussion

The incidence of phlebitis was 70% with use of peripheral intra venous catheters. It was higher than phlebitis rate reported in other studies, which ranged from 13–56% [4–6, 9–13, 15, 16]. The incidence of phlebitis was much higher than acceptable phlebitis rate recommended by Infusion Nurses Society (INS), which should be 5% or less [2]. High grade phlebitis (grade 3 and 4) accounted the majority (84%) based on Jackson’s Visual Infusion Phlebitis Scoring System, which required treatment and cannula re-site. Most indwelling catheters were detected after developing advanced stage of phlebitis, which might refer to ineffective preventive measures and poor catheter management. Phlebitis significantly occurred among those with catheter dwell time > 96 h as compared to catheter-in situ < 72 h (AOR = 2.261, 95% CI 1.087–4.702, P-value = 0.029). This finding was consistent with other studies [4, 6, 8–10, 13, 15]. Prolonged catheter dwell time predisposes for continued trauma by the catheter itself, longer contact to irritant drugs and infusates, and higher chance of exposure to bacterial colonization and infections. CDC guideline (2011) recommended routine replacement of PIVC no later than 96 h. There was lower rate of phlebitis among patients who used infusates (AOR = 0.472, 95% CI 0.280–0.796, P-value = 0.005) as compared to those who didn’t use infusates. This finding could be explained by frequent use of non-risky, isotonic solutions as infusates. Isotonic infusate has equal osmolality to that of blood, so then it might reduce phlebitis rate [8–10, 15]. Lower rate of phlebitis occurred among females (AOR = 0.293, 95% CI 0.031–0.626, P-value = 0.002). It was incongruent with reports from other studies, in which females outnumbered males in incidence of phlebitis [4, 7, 12, 16]. There was no sound explanation for gender-based differences regarding incidence of phlebitis.

### Limitation

Selection bias couldn’t be avoided as consecutive sampling method was used to recruit study subjects.

## Conclusion

The incidence of phlebitis was considerably high. High grade phlebitis (grade 3 and 4) occurred in the majority (84%) of patients with phlebitis. Catheter dwell time > 96 h was found to be a risk factor for increased incidence of phlebitis.

## Recommendation

The cannula must be reviewed on daily basis, and it should be removed if it stayed later than 96 h. Phlebitis protective measures and catheter management strategy should be improved at the study site.

## Data Availability

All data generated and analyzed are included in this research article.
